# *Rpv29*, *Rpv30* and *Rpv31*: Three Novel Genomic Loci Associated With Resistance to *Plasmopara viticola* in *Vitis vinifera*

**DOI:** 10.3389/fpls.2020.562432

**Published:** 2020-10-08

**Authors:** Maryam Sargolzaei, Giuliana Maddalena, Nana Bitsadze, David Maghradze, Piero Attilio Bianco, Osvaldo Failla, Silvia Laura Toffolatti, Gabriella De Lorenzis

**Affiliations:** ^1^Department of Agricultural and Environmental Sciences, University of Milan, Milan, Italy; ^2^Department of Agriculture and Life Sciences, Agricultural University of Georgia, Tbilisi, Georgia; ^3^Faculty of Viticulture and Winemaking, Caucasus International University, Tbilisi, Georgia; ^4^National Wine Agency of Georgia, Tbilisi, Georgia

**Keywords:** Georgia, GWAS, NB-LRR, SNP, Southern Caucasus, biotrophic plant pathogen, marker-assisted breeding

## Abstract

*Plasmopara viticola* (Berk. et Curt.) Berl. and de Toni, the agent of downy mildew, is one of the most important pathogens of European grapevine (*Vitis vinifera* L.). Extensive evaluation of cultivated grapevine germplasm has highlighted the existence of resistant phenotypes in the Georgian (Southern Caucasus) germplasm. Resistance is shown as a reduction in disease severity. Unraveling the genetic architecture of grapevine response to *P. viticola* infection is crucial to develop resistant varieties and reduce the impact of disease management. The aim of this work was to apply a genome-wide association (GWA) approach to a panel of Georgian-derived accessions phenotyped for *P. viticola* susceptibility and genotyped with Vitis18kSNP chip array. GWA identified three highly significant novel loci on chromosomes 14 (*Rpv29*), 3 (*Rpv30*) and 16 (*Rpv31*) associated with a low level of pathogen sporulation. *Rpv29*, *Rpv30*, and *Rpv31* loci appeared to be associated with plant defense genes against biotic stresses, such as genes involved in pathogen recognition and signal transduction. This study provides the first evidence of resistant loci against *P. viticola* in *V. vinifera* germplasm, and identifies potential target genes for breeding *P. viticola* resistant grapevine cultivars.

## Introduction

*Vitis vinifera* L. is one of the most widely cultivated fruit tree species of agricultural interest and it is the only species of the *Vitis* genus extensively used in the global wine industry. According to the data collected in 2018, viticulture covers approximately 7.6 million hectares worldwide and produces more than 67 million tons of grapes^1^. Unfortunately, *V. vinifera* is also known as the most susceptible *Vitis* species to *Plasmopara viticola* (Berk. et Curt.) Berl. and de Toni, the oomycete causing grapevine downy mildew. *P. viticola* was introduced into France from North America during the XIX century together with American wild *Vitis* species and rapidly spread across Europe dividing into two genetically distinct groups (Fontaine et al., 2013; Maddalena et al., 2020). Structure analysis indicated that the European and Italian *P. viticola* populations is formed by two separate genetic clusters, distributed according to a geographical gradient (East-West) and climatic conditions (Fontaine et al., 2013; Maddalena et al., 2020). *P. viticola* is a polycyclic pathogen able to biotrophically grow on tissues (leaves, shoots, and clusters) of susceptible *Vitis* species and, particularly, *V. vinifera*. If adequate disease management strategies are not applied, the disease seriously affects yield in terms of on grape quality and quantity (Toffolatti et al., 2018b).

Resistant accessions within the North American *non-vinifera* species, such as *Vitis riparia* Michx., *Vitis cinerea* (Engelm. ex A.Gray) Engelm. ex Millard and *Vitis labrusca* L., and the Northeast Asian species (*Vitis amurensis* Rupr.), exhibit varying levels of resistance, ranging from moderate to high, due to co-evolution with the pathogen (Jürges et al., 2009). Several QTL (Quantitative Trait Loci), conferring downy mildew resistance, at different levels ranging from weak to total, were discovered in *Vitis* species background: *Rpv1* and *Rpv2* in *Muscadinia rotundifolia* Michaux (Merdinoglu et al., 2003; Wiedemann-Merdinoglu et al., 2006); *Rpv3* and *Rpv19* in *Vitis rupestris* Scheele (Welter et al., 2007; Bellin et al., 2009; Divilov et al., 2018; Vezzulli et al., 2019; Foria et al., 2020); *Rpv4*, *Rpv7*, *Rpv11*, *Rpv17*, *Rpv18*, *Rpv20*, and *Rpv21*, in unspecified American species (Fischer et al., 2004; Welter et al., 2007; Bellin et al., 2009; Divilov et al., 2018); *Rpv5*, *Rpv6*, *Rpv9*, and *Rpv13* in *V. riparia* (Marguerit et al., 2009; Moreira et al., 2011); *Rpv8*, *Rpv10*, *Rpv12*, *Rpv22*, *Rpv23*, *Rpv24*, *Rpv25*, and *Rpv26* in *V*. *amurensis* (Blasi et al., 2011; Schwander et al., 2012; Venuti et al., 2013; Song et al., 2018; Lin et al., 2019); *Rpv14* in *V. cinerea* (Ochssner et al., 2016); *Rpv15* and *Rpv16* in *Vitis piasezkii* Maxim. (Pap et al., unpublished); *Rpv27* in *Vitis aestivalis* Michx. (Sapkota et al., 2015, 2019); and *Rpv28* (Bhattarai et al., in preparation; www.vivc.de).

The management of downy mildew on traditional *V. vinifera* varieties requires regular application of fungicides. It is estimated that in the European Union, viticulture accounts for approximately 70% of all agrochemicals used, most of which are applied to contain the agents of downy and powdery mildews. Nevertheless, the intensive use of chemicals is becoming more and more restricted because of their high costs, their risks to human health and their negative environmental impact due to the chemical residues detected in grapes, soil and aquifers. In addition, disease control could be difficult to attain in future because some *P. viticola* strains could develop site-specific fungicide resistances, leading to great difficulties in the management of disease, while the discovery of new modes of action is rare (Hollomon, 2015). The EU Directive 2009/128 for sustainable management of diseases caused by plant pathogens in Europe strongly recommends a reduction in the number of treatments in the field. Moreover, the application of Regulation 1107/2009, concerning the placement on the market of plant protection products, is causing a reduction in the active substances available. The exploitation of resistance sources is the best way to decrease the use of chemicals for disease management and to achieve an effective protection from *P. viticola* in an environmental friendly way. Breeders had already started crossing the susceptible *V. vinifera* varieties with American species in the XIX century, first in United States and then in Europe (Eibach and Töpfer, 2015; Migicovsky et al., 2016; Merdinoglu et al., 2018; Yobrégat, 2018). Nowadays, numerous varieties combining resistance traits from American and Asian species and the quality traits of *V. vinifera* are available (Reynolds, 2015). A comprehensive list of new resistant varieties can be accessed from the *Vitis* International Variety Catalog website (VIVC; www.vivc.de).

Finding new sources of resistance is of paramount importance in breeding for biotic stress resistance in a perennial crop, which has to be productive for years while maintaining its resistance characteristics at the same time: the main strategy for preventing the selection of pathogen strains able to overcome resistance is, in fact, pyramiding resistance genes in the crop variety (Eibach et al., 2007; Delmotte et al., 2016; Zini et al., 2019). Recently, unique resistance traits to the downy mildew agent have been reported in *V*. *vinifera* varieties (Bitsadze et al., 2015; Toffolatti et al., 2016) coming from the first domestication center of the species: Georgia, Southern Caucasus (Imazio et al., 2013). The resistance mechanism for one of these resistant cultivars, named Mgaloblishvili, has been studied in detail (Toffolatti et al., 2018a, 2020). After artificial inoculation, *P. viticola* growth and sporulation are significantly affected in Mgaloblishvili: the mycelium degenerates, sporangiophores show an altered morphology and lower numbers of sporangia are produced, without any evidences of the hypersensitive response that occurs in American species. From the transcriptomic point of view, its defense mechanism shows an overexpression of genes related to pathogen recognition through PAMP (pathogen-associated molecular patterns), DAMP (damage-associated molecular patterns), and effector receptors and ubiquitination, signaling pathway through ethylene, synthesis of antimicrobial compounds (such as monoterpenes and flavonoids) and fungal wall degrading enzymes, and the development of structural barriers (such as cell wall reinforcement). The discovery of resistance to *P. viticola* in *V. vinifera* promises fresh opportunities for grapevine breeding in terms of new resistant loci.

Breeding for disease resistance is a very time-consuming process (up to 25–30 years are required for a breeding program), because it needs the evaluation of resistance levels of the progeny and other important characteristics (yield and quality of vines), which are typically not achieved until the third year after planting. A way to considerably decrease the length of the breeding process (accelerating the process by up to 10 years) is the adoption of the marker-assisted selection (MAS) approach, which allows the targeted selection of progeny harboring the resistance loci (Eibach and Töpfer, 2015).

Identification of genomic loci associated with complex quantitative and qualitative traits was enabled by the development of QTL (quantitative trait locus) and GWA (genome wide association) mapping approaches, combining genetic and phenotypic data. QTL mapping is performed using segregating biparental populations, while GWA approach relies on historical recombination events which occurred in natural populations, germplasm collections and breeding materials (Korte and Farlow, 2013). Over the last 10 years, NGS (next-generation sequencing) technologies have made available numerous (from thousands to hundreds of thousands) SNP (single nucleotide polymorphism) markers to be used for GWA study (GWAS) in various plant and animal species (Bhat et al., 2016).

In grapevine, at least three high-density SNP arrays have been set up (Myles et al., 2010; Marrano et al., 2017; Laucou et al., 2018), and the most used SNP set is the Vitis18kSNP chip array, developed by the GrapeReSeq Consortium, re-sequencing the genome of 47 *V*. *vinifera* genotypes and 18 genotypes belonging to American *Vitis* species and holding 18,071 SNPs. This high-density SNP array has been demonstrated to be a valid method for mapping of both quantitative and qualitative traits (Laucou et al., 2018).

In the present work, the Vitis18kSNP chip array was used to genotype a panel of *V. vinifera* Georgian accessions to identify genomic regions and/or putative markers associated with *P. viticola* resistance in *V. vinifera*, through a GWA approach, to be used for MAS in further breeding programs.

## Materials and Methods

### Plant Materials

**FIGURE 1:**
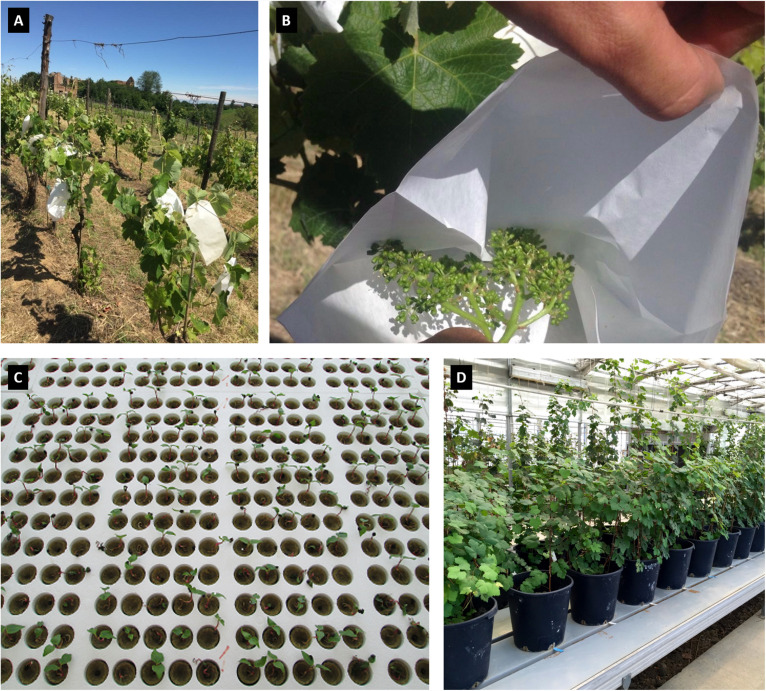
Some stages of Mgaloblishvili self-pollination **(A,B)**, seedling germination **(C)** and plant maintenance in greenhouse **(D)**.

The panel of accessions analyzed in this study (Supplementary Table 1) accounted for 132 genotypes: 84 are seedlings of the Mgaloblishvili self-pollinated population, and 48 are genotypes belonging to the Georgian germplasm collection. The breeding-derived genotypes are maintained in the greenhouse of the Department of Agricultural and Environmental Sciences (DiSAA), located in Arcagna (Lodi, Italy) and the germplasm genotypes are planted in the DiSAA germplasm collection vineyard, located in Torrazza Coste (Pavia, Italy). Mgaloblishvili self-progeny was obtained in spring of 2012, by enclosing Mgaloblishvili inflorescences in paper bags before flowering. At harvesting, bunches were collected and the seeds were extracted from berries to be vernalized at 5°C for 2 months in humid sand. The vernalized seeds were placed in plates of polystyrene cups filled with rockwool and maintained at 20 to 25°C up to germination in a screenhouse. The seedlings were transplanted into 8-cm pots filled with a sand–peat mixture (7:3 in volume) and after 1 year were moved in 20-cm pots. The plants were regularly irrigated and maintained without mineral fertilization practice. In Figure 1, some stages of Mgaloblishvili self-pollination, seedling germination and plant maintenance in greenhouse are shown.

### Phenotyping

The degree of susceptibility to *P. viticola* was evaluated through experimental inoculation on leaf samples collected at the beginning of the 2015, 2016, and 2017 grapevine growing seasons, using the protocol described by Toffolatti et al. (2016). To maximize the genetic variability of the pathogen and allow the detection of accessions that were resistant to a wide range of pathogen strains, field populations of *P. viticola* were used for the experimental inoculations (Toffolatti et al., 2016). Recent studies demonstrated that the European and Italian *P. viticola* population is divided in two genetic clusters, separated over an east-west gradient (Fontaine et al., 2013; Maddalena et al., 2020). In this study, east and west populations of *P. viticola* coming from Italy, at S. Maria della Versa (Pavia; East population) and Casarsa della Delizia (Pordenone; West population), and Georgia (West), were mixed to perform experimental inoculations. Phenotypical evaluations were performed in triplicate. Briefly, three leaf discs (1.5 cm in diameter) were cut from three leaves collected from the 3rd–5th leaf starting from the shoot apex of the plants. The leaf disks were sprayed with 1 mL *P. viticola* sporangia suspension (5 × 10^4^ sporangia⋅mL^–1^) and incubated in a humid chamber at 22°C for 10 days. Disease severity was estimated from the area covered by sporulation by calculating the Percentage Index of Infections (I%I) (Townsend and Heuberger, 1947; Toffolatti et al., 2016). The accessions with an average I%I lower than 25% along the three sampled years were considered resistant. The 25% threshold was chosen based on the I%I distribution. Box plot distribution of the three replicate values of the samples showed that only nine samples (ID 124, ID 122, ID LIB 56, ID 138, ID 109, ID L22A, ID M22F, ID M22A, ID M22E) showed I%I < 40%, while the others reached higher values (Supplementary Figure 1). The average I%I of these samples was 20 ± 5% (95% confidence interval). Therefore, 25% was the chosen threshold. The existence of differences between I%I recorded in different years was analyzed by Pearson’s correlation coefficient.

Resistance levels (RLs), expressed in percentage, were calculated for each accessions by using the following formula:

$RL=100-(\frac{I\%\,I_{x}}{I\%\,I_{M\,A\,X}}\times 100$) where I%I_x_ is the average disease severity of the sample and I%I_MAX_ is the maximum value of disease severity recorded (accession ID 157 M, I%I = 85.8%).

### SNP Genotyping

The 132 genotypes were genotyped using the Vitis18kSNP array (Illumina Inc., San Diego, CA, United States), containing 18,071 SNPs. The genotyping of breeding-derived accessions (Mgaloblishvili seedlings) was performed in this work, while for germplasm genotypes the data were obtained by De Lorenzis et al. (2015). Genotyping was carried out on 200 ng of genomic DNA extracted from 100 mg of frozen young leaf tissue using NucleoSpin^®^ Plant II (MACHEREY-NAGEL, Germany), according to the manufacturer’s protocol. DNA concentration and quality were checked by electrophoresis on agarose gel and by spectroscopy using a NanoDrop Spectrophotometer (Thermo Fisher Scientific, Waltham, MA, United States) and Quant-iT dsDNA HS assay kit for Qubit 3.0 Fluorometer (Thermo Fisher Scientific). Genotyping analysis was performed by the laboratory of Fondazione Edmund Much (San Michele all’Adige, Trento, Italy).

### Data Analysis

SNP data produced in this work (84 Mgaloblishvili seedlings) were filtered for samples showing a call quality value (p50GC) lower than 0.54 and loci with a GenTrain (GT) score value lower than 0.6 and a marker missing rate > 20% (De Lorenzis et al., 2015). The Mgaloblishvili self-pollinated population dataset and the SNP profiles of 48 varieties reported in De Lorenzis et al. (2015) were merged and filtered for minor allele frequency (MAF) > 5%.

MEGA 7.0 software (Kumar et al., 2016) was used to design a UPGMA (Unweighted Pair Group Method with Arithmetic Mean) phylogenetic tree, based on the Dice’s coefficient (Dice, 1945) distance matrix generated by PEAS 1.0 software (Xu et al., 2010). Principal Component Analysis (PCA) was carried out using *adegenet* package (Jombart, 2008) of R software (R Core Team), and the first two components values were plotted on a 2-D scatterplot. Structure analysis was carried out using LEA package (Frichot and François, 2015) of R software by varying the number of ancestral genetic groups (K) from 1 to 10 in ten repetition runs for each *K* value. The most likely *K* value was detected based on LEA cross-validation method.

The LD (linkage disequilibrium) estimation as Pearson’s squared correlation coefficient (r^2^) between each pair of molecular markers (Zhao et al., 2005) was evaluated using PLINK (Purcell et al., 2007) software. The pair-wise LD as r^2^ was calculated using the parameters –ld-window-r2 0,–ld-window 99999,–ld-window-kb 10000. The distances between loci were categorized into intervals of a fixed length (100 kb) and, for each interval, average r^2^ was calculated. The LD decay was visualized by plotting the average r^2^ per each interval from 0 up to 10 Mb by R software.

Association analysis was performed in R software using GAPIT package (Lipka et al., 2012). GLM (Generalized Linear Model), MLM (Mixed Linear Model), MLMM (Multiple Locus Mixed linear Model), FarmCPU (Fixed and random model Circulating Probability Unification) and SUPER (Settlement of MLM Under Progressively Exclusion Relationship) algorithms were tested. For fixed effect, Q-matrix (for *K* = 3), detected by LEA, was used as the covariate for association analysis accounting for population structure. The GWA algorithm performances were evaluated through quantile-quantile (QQ) plots. A conservative threshold for assessing SNP significance was calculated based on Bonferroni correction for a type I error rate of 0.05. The SNPs fitting a logistic regression, performed in PLINK software, were selected.

### Candidate Gene Mining

Gene associated with SNP loci passing the Bonferroni-adjusted threshold were predicted based on the LD r^2^ threshold of 0.2 (Li et al., 2014), using the grapevine reference genome PN40024 (12X.v2 version) (Canaguier et al., 2017). The SNP loci mapping to reference genome was conducted using CLC Genomic Workbench software (v. 20.0) in advance sequence finder toolbox including negative strand. Nearby genes in linkage regions of stable SNP-trait associations with putative functions supposedly related to the *P. viticola* resistance trait were selected as candidates.

## Results

### Phenotypic and Genetic Diversity of Accession Panel

**FIGURE 2:**
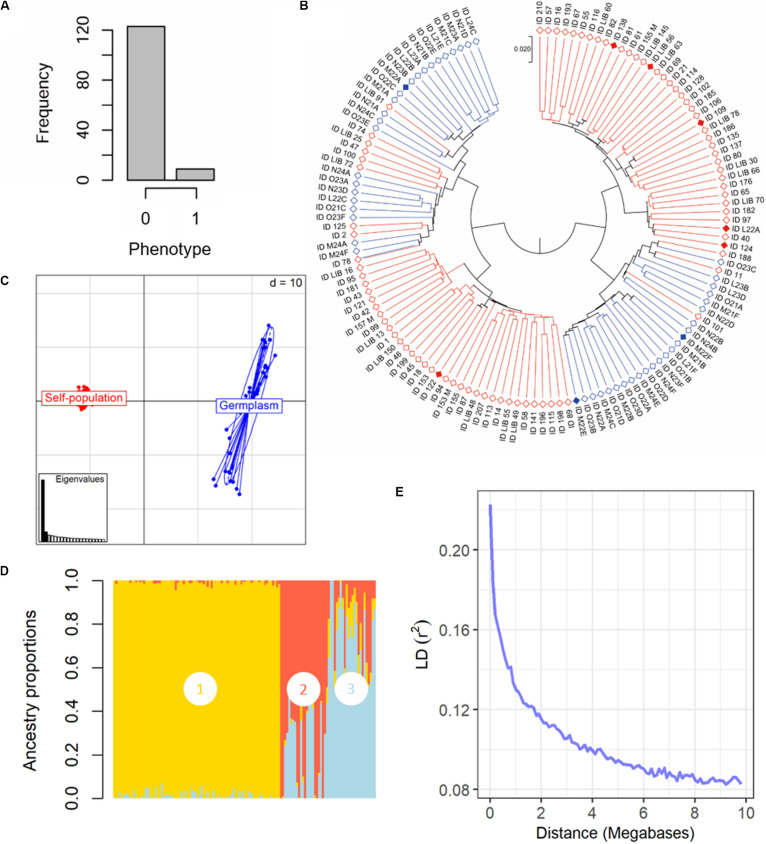
Phenotypical and genetic diversity in the panel of 132 grapevine accessions, belonging to the Mgaloblishvili self-pollinated population (84) and Georgian germplasm population (48), used for GWA analysis. The individuals were phenotyped for resistance to *P. viticola* infection and were genotyped using the Vitis18kSNP array. **(A)** Histogram summarizing the frequency of susceptible (0) vs. resistant (1) phenotypes. **(B)** UPGMA dendrogram showing relationships among individuals of Mgaloblishvili self- pollinated (red) and Georgian germplasm population (blue). Filled rhombus indicate resistant accessions **(C)** Scatterplot relationships among individuals of Mgaloblishvili self- pollinated (red) and Georgian germplasm population (blue), as represented by the first two principal components (PC1 along the horizontal axis, PC2 along the vertical axis) of PCA. **(D)** Admixture proportions as estimated by LEA package at *K* = 3, displayed in a barplot. Each sample is represented as a vertical bar, reflecting assignment probabilities to each of the three groups. Group 1: Mgaloblishvili self-pollinated individuals. Group 2 and 3: Georgian germplasm population individuals. **(E)** Decay of average linkage disequilibrium (LD r^2^) over distance (Mb).

Phenotyping evaluations were performed for 3 years (2017–2019) and only genotypes scored with a I%I < 25% in the 3 years of evaluation were classified as resistant. Evaluation trials have shown an overall high susceptibility to *P. viticola* infection, with some accessions showing a large distribution of the data (Supplementary Figure 1). Nine out of 132 genotypes were resistant: five Mgaloblishvili seedlings (ID 124, 122, LIB 56, 138, 109), Mgaloblishvili and three varieties (Jani Bakhvis, Zerdagi, and Kamuri shavi) (Figure 2A and Supplementary Table 1). The samples showed a significant correlation among years (*r* > 0.991; *N* = 3; *P* < 0.043). RLs of the nine resistant genotypes ranged from 70 to 84% (Supplementary Table 1). None of the resistant genotypes showed HR in leaf tissues.

The SNP genotyping data of the Mgaloblishvili self-pollinated population were merged with the ones of 48 Georgian cultivars (De Lorenzis et al., 2015). The final dataset accounted for 132 genotypes and 12,825 SNP loci (Supplementary Table 2). Clustering analysis discriminated the genotypes in two well distinct main groups (Figure 2B). In each main group, both breeding-derived genotypes and germplasm cultivars were included, though they were mainly clustered in well separated sub-groups. Resistant genotypes were distributed between the two main groups. The range of identity varied from 95 to 88%. PCA strongly differentiated Mgaloblishvili self-pollinated and germplasm individuals into two distinct groups (Figure 2C). The first two principal components (PCs) captured 33% of total explained variance (PC1 = 29% and PC2 = 4%). The two groups were separated along the PC1. As expected, the germplasm individuals showed a variability higher than the breeding-derived accessions. According to the cross-validation plot, structure analysis identified three ancestral populations (*K* = 3), one for Mgaloblishvili seedlings (group 1) and two for germplasm individuals (groups 2 and 3) (Figure 2D). The three resistant cultivars were assigned one to group 2 (Zerdagi) and two to group 3 (Jani Bakhvis and Kamuri shavi). The percentage of admixed genotypes (with a membership probability <80%) was 28%. All the admixed genotypes were detected among the cultivars (Supplementary Table 3). All the nine resistant genotypes showed a membership probability higher than 80%. LD decay was estimated for the entire dataset (Figure 2E). LD decreased with the increase in physical distance between marker loci. Average LD decay (*r*^2^ = 0.11) was observed after ∼2 Mb. The LD value dropped to 0.2 after ∼100 kb.

### GWA Analysis

**FIGURE 3:**
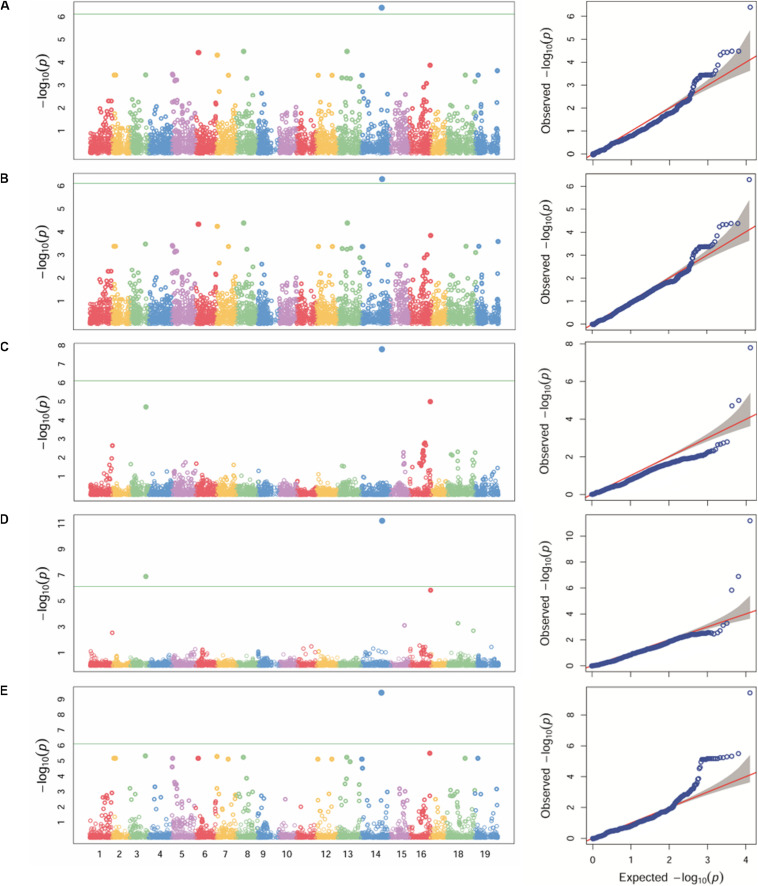
Manhattan plot (left) of -log_10_ *p*-values estimated for binary (resistant vs. susceptible) coded phenotypic response to *P. viticola* infection in the panel of 132 accessions genotyped by 18 k SNPs. Significant SNPs are circles above the Bonferroni-adjusted threshold (green horizontal line). Quantile-quantile plot (right) of expected vs. observed -log_10_ *p*-values. Association analysis results of GLM **(A)**, MLM **(B)**, MLMM **(C)**, FarmCPU **(D)** and SUPER **(E)** algorithms.

Different statistical models (GLM, MLM, MLMM, FarmCPU and SUPER) were tested for detecting associations for *P. viticola* resistance. Because structure analysis was able to capture the differences among the Georgian germplasm cultivars better than PCA, Q-matrix for *K* = 3 was used as covariate in the GWA analysis. The application of GLM, MLM and SUPER models allowed to account for stratification, although a relevant number of false positives was detected (Figures 3A,B,E). A significant SNP associated with *P. viticola* infection was identified in the three tested models: the SNP (chr14_21613512_C_T) located in the chromosome 14 at position 21,613,512 with a *p*-value of 4.01e-07, 5.09e-07 and 3.68e-10, respectively, for GLM, MLM and SUPER models. MLMM and FarmCPU models reduced false positive associations (Figures 3C,D). MLMM models detected one significant SNP associated with *P. viticola* infection, with a -log_10_ *p*-value above the Bonferroni-adjusted threshold, and two SNPs below the Bonferroni-adjusted threshold. The first SNP was the same detected by the GLM, MLM and SUPER models, with a *p*-value of 1.25e-08. The remaining two SNPs were li_T_C_chr16_21398409, located on chromosome 16 at position 21,398,409 and a *p*-value of 7.9e-06 and cn_C_T_chr3_16229046, located on chromosome 3 at position 16,229,046 and a *p*-value of 1.25e-05. FarmCPU model detected the same SNPs detected by MLMM model. chr14_21613512_C_T and cn_C_T_chr3_16229046 were above the Bonferroni-adjusted threshold, with *p*-values of 8.23e-08 and 8.18e-04, respectively, while li_T_C_chr16_21398409 was slightly below the threshold, with a *p*-value of 6.25e-03.

For an approximate estimation of allelic effect, a logistic regression was fitted for the three significant SNPs. As observed by odds ratio, highly significant association was confirmed for chr14_21613512_C_T locus, followed by li_T_C_chr16_21398409 and cn_C_T_chr3_16229046 (Table 1).

**TABLE 1:** Allelic effect estimation by logistic regression for SNP loci associated with *P. viticola* resistance traits.

**SNP ID**	**Chromosome**	**Genome position (bp)**	**Odds ratio**	***p*-value**
chr14_21613512_C_T	14	21,613,512	28.39	0.00021
cn_C_T_chr3_16229046	3	16,229,046	3.74	0.00143
li_T_C_chr16_21398409	16	21,398,409	7.33	0.00179

*Odds ratio and *p*-values are reported.*

### Candidate Gene Prediction

The three SNP loci passing the Bonferroni-adjusted threshold were mapped to *V. vinifera* reference genome (PN40024 12X) to identify putative genes related to the *P. viticola* resistance trait. The LD value (r^2^) dropped to 0.2 after ∼100kb, for this reason a window of 100 kb upstream and downstream the most significant SNPs was chosen to search for candidate genes. Supplementary Table 4 reports the list of candidate genes in a window of 100 kb upstream and downstream the three SNPs associated to *P. viticola* resistance trait. Supplementary Table 5 reports the SNP allele information associated to these three regions.

**FIGURE 4:**
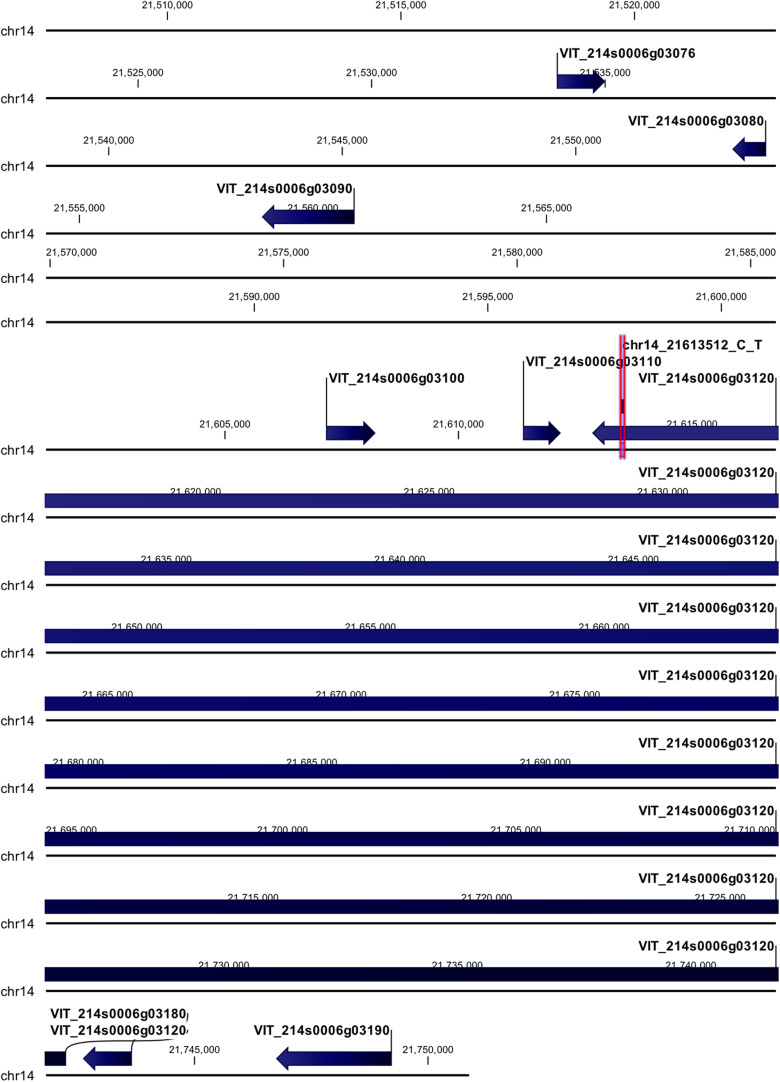
Annotation on grapevine reference genome PN40024 of chr14_21613512_C_T locus (*Rpv29*), on chromosome 14, associated with *P. viticola* resistance trait in *V. vinifera*. Locus is indicated with red arrows/violet bar, genes are indicated with blue arrows.

**FIGURE 5:**
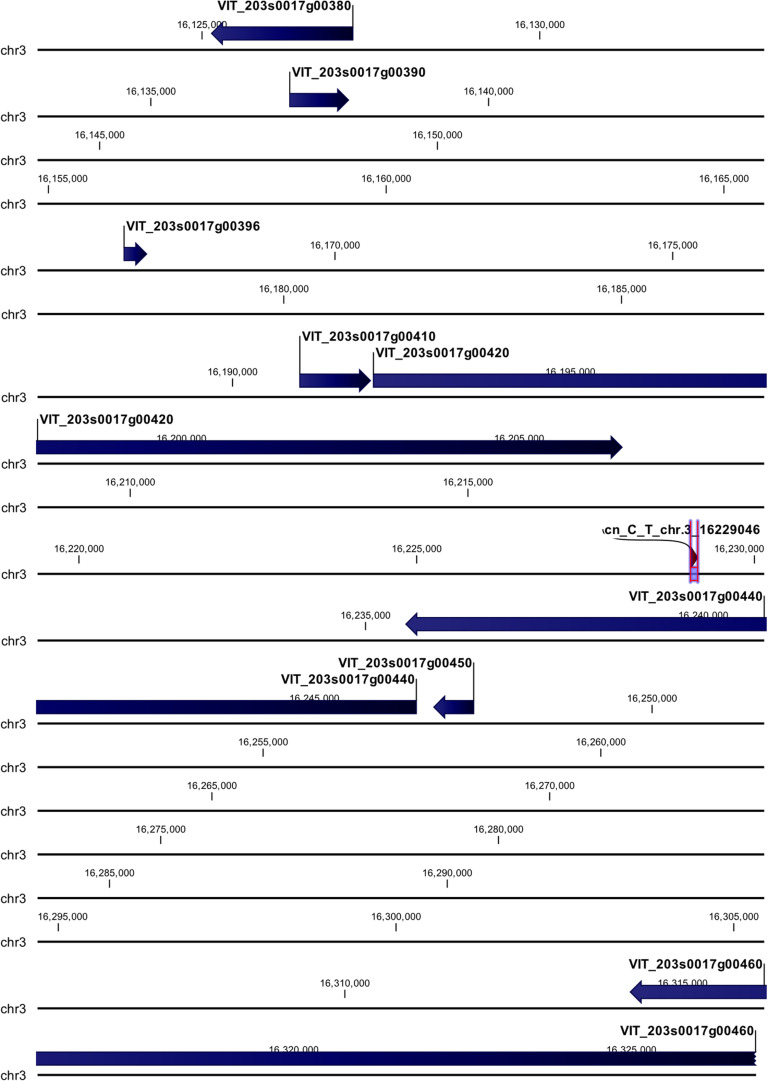
Annotation on grapevine reference genome PN40024 of cn_C_T_chr3_16229046 locus (*Rpv30*), on chromosome 3, associated with *P. viticola* resistance trait in *V. vinifera*. Locus is indicated with red arrows/violet bar, genes are indicated with blue arrows.

**FIGURE 6:**
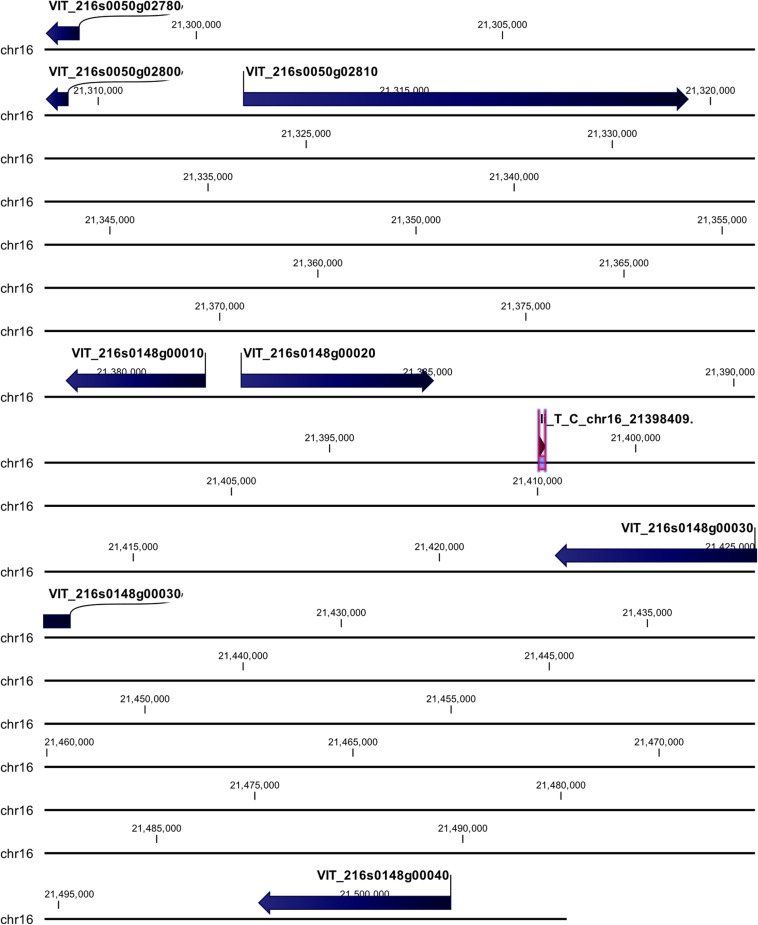
Annotation on grapevine reference genome PN40024 of li_T_C_chr16_21398409 locus (*Rpv31*), on chromosome 16, associated with *P. viticola* resistance trait in *V. vinifera*. Locus is indicated with red arrows/violet bar, genes are indicated with blue arrows.

The chr14_21613512_C_T locus mapped in the coding region of HEAT repeat-containing 5B protein (VIT_214s0006g03120) (Figure 4). The polymorphism (G → A) was non-synonymous giving rise to a change in the encoded amino acid, from aspartic acid (D) to asparagine (N). Upstream of this locus five genes were annotated: three of them encode for uncharacterized proteins (VIT_214s0006g03076, VIT_214s0006g03080, and VIT_214s0006g03100), and two for a probable cellulose synthase A catalytic subunit 8 [UDP-forming] (VIT_214s0006g03090) and an acyl-CoA-binding domain-containing protein 3-like (VIT_214s0006g03110). Downstream of this locus two genes were annotated, encoding for a probable carboxylesterase 17 and a plant cadmium resistance 4 protein (VIT_214s0006g03180 and VIT_214s0006g03190, respectively).

cn_C_T_chr3_16229046 and li_T_C_chr16_21398409 loci were mapped in intragenic regions (Figures 5, 6). The first locus was localized in a region including, upstream, an uncharacterized protein (VIT_203s0017g00420), a magnesium-dependent phosphatase 1 (VIT_203s0017g00410), an ubiquitin carboxyl-terminal hydrolase 21 (VIT_203s0017g00396), a MADS-box protein JOINTLESS-like (VIT_203s0017g00390), and a magnesium-dependent phosphatase 1-like (VIT_ 203s0017g00380), downstream, an uncharacterized protein (VIT_203s0017g00440), a MADS-box protein JOINTLESS-like (VIT_203s0017g00450) and an inositol transporter 1 (VIT_ 203s0017g00460). The second locus mapped in the genomic region including, upstream, two rust resistance kinase Lr10-like genes (VIT_216s0148g00020, VIT_216s0148g00010) and two genes encoding for uncharacterized proteins (VIT_216s0050g02810, VIT_216s0050g02800), and downstream, two rust resistance kinase Lr10-like genes (VIT_216s0148g00030 and VIT_216s0148g00040).

## Discussion

Downy mildew is one of the most important diseases affecting grapevines worldwide. So far, the sources of resistance were searched for in non-*vinifera* species, such as *V. labrusca*, *V. aestivalis*, *V. riparia*, *V. rotundifolia* and *V. amurensis*. The identification of resistant cultivars in the *V. vinifera* Georgian germplasm gave us the possibility to explore this promising material. In this work, a first insight was provided into quantitative resistance loci affecting downy mildew resistance traits in *V. vinifera* using an association mapping approach.

### Grapevine Resistant Cultivars Belong to Different Georgian Regions

Experimental inoculations on 132 grapevine individuals belonging to the Mgaloblishvili seedling population and Georgian germplasm confirmed the high susceptibility of *V. vinifera* to *P. viticola* infection. Almost all breeding-derived and germplasm accessions were severely affected by the pathogen, developing medium to high I%I. Several accessions showed a large variability in the I%I distribution: this variability is frequently occurring in field assessment and in bioassays (Cadle-Davidson, 2008; Calonnec et al., 2013; Toffolatti et al., 2016) and could be related to several factors among which are the physiological state of the plant and the virulence of the pathogen. It is due to this variability that the experimental inoculations have been carried out in different years with mixed inocula: to identify those accessions that consistently showed a resistant behavior. A limited number of accessions (five breeding-derived and four germplasm accessions) clearly showed a reduced disease severity, which ranged from 5 to 25%. None of the accessions showed any necrotic spots, which are associated with HR, confirming that the defense mechanism is different from the one observed for North American and Asian *Vitis* species (Toffolatti et al., 2018a; Dry et al., 2019). The resistant cultivars showed different genetic origins. They were grouped in two different clusters and ancestral groups, characterized by cultivars having the same geographical provenance (Imazio et al., 2013; De Lorenzis et al., 2015). Zerdagi, a variety originated from Samegrelo province in the Western Georgia, was grouped with cultivars coming from Southern regions and Jani Bakhvis and Kamuri shavi with cultivars coming from the Western and Eastern regions.

### Multi-Locus GWA Models Are the Best for Studying Complex Traits

The GWA approach was applied by genotyping 132 grapevine individuals with the 18k SNP genotyping array. A recent study has demonstrated the power of this array in detecting both known (such as berry color) and novel (such as acidity) loci related to phenotypic traits *via* GWA (Laucou et al., 2018). GWAS requires a genomic map with a marker density higher than the LD extent (Brachi et al., 2011). In our dataset, the average LD declined with the increase of the physical distance between markers, as already estimated in grapevine (Myles et al., 2010; Laucou et al., 2018; Figure 2E). The high LD levels observed in grapevine and the average inter-SNP spacing (about one SNP every ∼47 kbp, (Laucou et al., 2018), appear to be enough to tag associated loci.

Regarding the accuracy, the effectiveness of the GWA approach is strongly influenced by population stratification. Breeding-derived and germplasm accessions clearly showed genetic differentiation (Figures 2B–D). Since structure analysis was better able than PCA to capture the level of stratification, structure results were used as covariates for association analysis. Accounting for the complexity of phenotypic dataset and known population stratification, different algorithms, both single- (GLM, MLM, and SUPER) and multi-locus (MLMM and FarmCPU), for modeling marker-trait associations were tested. It is widely accepted that multi-locus GWAS models are superior to single-locus GWAS methods to identify association (Cui et al., 2018). In our study, multi-locus GWAS models detected the highest number of significant SNPs: FarmCPU = two (plus one just above the Bonferroni-adjusted threshold; MLMM = one (plus two Bonferroni-adjusted threshold); GLM, MLM and SUPER = one (Figure 3). Furthermore, our results confirm the usefulness of the *Vitis* SNP genotyping array in detecting loci associated with phenotypical traits (Laucou et al., 2018).

### Three Novel SNP-Trait Associations to *P. viticola* Resistance Were Identified

To date, up to 28 QTL conferring resistance to downy mildew have been identified within wild *Vitis* species (Dry et al., 2019; www.vivc.de), but only two, *Rpv1* and *Rpv3* were characterized (Feechan et al., 2013; Eisenmann et al., 2019), mapping on chromosomes 12 and 18, respectively. *Rpv1* is a NB-LRR (nucleotide-binding site leucine-rich repeat) receptor, while *Rpv3* is associated with the biosynthesis of stilbenes. In our study, clear signals were identified on chromosomes 14, 3, and 16. The signal on chromosome 14, related to chr14_21613512_C_T locus, was recorded in all the five tested models, while the other two, related to cn_C_T_chr3_16229046 and li_T_C_chr16_21398409, were recorded only in MLMM and FarmCPU models, with some differences in the *p*-value. Among the 27 QTL already identified, three (*Rpv8*, 12, and 19) map on chromosome 14, while no QTL were found to map on chromosomes 3 and 16. *Rpv8* and *Rpv12* both mapped on the upper arm of chromosome 14 (Blasi et al., 2011; Venuti et al., 2013), while *Rpv19* mapped on the lower arm, at around position 24 Mb (Divilov et al., 2018). Because the SNPs identified in this work do not physically co-locate to the QTL already identified, it is possible to conclude that the three loci are novel associations. We designated the locus on chromosome 14 (for chr14_21613512_C_T) *Rpv29*, the locus on chromosome 3 (cn_C_T_chr3_16229046) *Rpv30* and the locus on chromosome 16 (li_T_C_chr16_21398409) *Rpv31*.

The logistic regression values (Table 1) indicated that the *Rpv29* locus is the one having a major effect on the phenotype. Furthermore, the other two loci, *Rpv30* and *Rpv31*, showed a statistically significant *p*-value as well, although the allelic effect estimation is lower. Nevertheless, since the resistance mechanism of accessions analyzed in this work did not show HR, it suggests that more than one locus are necessary to acquire the resistance.

### *Rpv29*, *Rpv30* and *Rpv31* Are Markers Associated With Genes Related to *P. viticola* Resistance in *V. vinifera*

NB-LRR genes appeared to be associated with *Rpv12* locus in the upper arm of chromosome 14 (Venuti et al., 2013). The SNP located on chromosome 14 (*Rpv29*) mapped in the coding region of HEAT repeat-containing 5B protein and the polymorphism leads to a non-synonymous amino acid substitution from aspartic acid to asparagine. Further studies are needed to better understand the effect at the protein level. HEAT motifs are tandemly repeated sequences of about 50 amino acid residues identified in a wide variety of eukaryotic proteins (Andrade et al., 2001). It was demonstrated that repeat proteins possess an intrinsic ability to bind peptides, acting as an integral component of protein complexes (Sharma and Pandey, 2016). HEAT repeat proteins, such as ILA, are required for plant immunity. In *Arabidopsis thaliana*, ILA is required for both non-host and basal resistance against *Pseudomonas syringae*, for resistance mediated by NB-LRR proteins and for systemic acquired resistance (SAR) (Monaghan and Li, 2010). NB-LRR proteins act as specific receptors of pathogen effectors, activating defense mechanisms leading to effector-triggered immunity (ETI) (Jones and Dangl, 2006). It is therefore tempting to speculate that the chr14_21613512_C_T locus could be involved in primary plant-pathogen interactions leading to both ETI and SAR. Nevertheless, further investigations are needed to confirm this result.

In a region spanning ∼100 kb upstream and downstream the *Rpv29* locus, four genes, encoding for a probable cellulose synthase A catalytic subunit 8 [UDP-forming], an acyl-CoA-binding domain-containing protein 3-like, a probable carboxylesterase 17 and a plant cadmium resistance 4 protein, as well as three genes encoding for uncharacterized proteins, were mapped. All the candidate genes, except plant cadmium resistance 4 protein, appeared to be related to plant defense mechanism, based on the literature.

Cellulose synthases are involved in the secondary cell wall formation (Taylor et al., 2000). Structural modification, such as cell wall thickening, is one of the mechanisms adopted by plants to contrast the pathogen infection (Schulze-Lefert, 2004). A number of evidences proving the connection between cell wall structure and stress signaling, leading to enhanced production of hormones (such as jasmonate and ethylene) and to enhanced resistance to a broad range of pathogens were described (Ellis and Turner, 2001). Similarly to other organisms, Mgaloblishvili showed an up-regulation of genes, such as cellulose synthase-like protein G3 gene, that are involved in the transition from primary to secondary wall synthesis (Taylor et al., 1999).

Acyl-CoA binding proteins are thought to facilitate the transport of fatty acids/lipids among the cells (Kragelund et al., 1993). They are required for PAMP resistance to fungal pathogens, as described for *A. thaliana* against *Botrytis cinerea* and *Colletotrichum higginsianum* (Xia et al., 2012).

Carboxylesterases (CXEs) are a large family of enzymes, belonging to the α/β hydrolase fold superfamily, that hydrolyze ester, amide, and carbamate bonds (Putterill et al., 2003). They are involved in plant defense responses. *Nicotiana tabacum*, *A. thaliana*, and *Capsicum annuum* showed some CXEs involved in the plant-pathogen interaction, some of them related to hypersensitive response (Pontier et al., 1994; Kim et al., 2001; Putterill et al., 2003). In *Vitis flexuosa*, some CXEs were upregulated in response to *Botrytis cinerea*, *Elsinoe ampelina*, and *Rhizobium vitis* infection, indicating a putative role in defense mechanism during pathogen infection (Islam and Yun, 2016).

The cn_C_T_chr3_16229046 locus on chromosome 3 (*Rpv30*) was annotated close to predicted genes, such as MADS-box protein JOINTLESS-like, ubiquitin carboxyl-terminal hydrolase 21, magnesium-dependent phosphatase 1 and 1-like (MDP-1 and MDP-1-like) and inositol transporter 1 (INT1), and two genes encoding for uncharacterized proteins. All the candidate genes, except INT1, appeared to be related to plant defense mechanism, based on the literature.

MADS-domain transcription factors are proteins involved in multiple developmental pathways in plants, animals, and fungi (Castelán-Muñoz et al., 2019). JOINTLESS is a MADS-domain transcription factor, that together with MACROCALYX, induces the expression of AP2/ERF (ethylene response factor) 52 transcription factor in tomato during pre-abscission and abscission stages of pedicel (Nakano et al., 2014). Transcriptional data revealed that Mgaloblishvili defense mechanism is mediated mainly by ethylene (Toffolatti et al., 2018a). This MADS-domain transcription factor can be related to *P. viticola* resistance mechanism in *V. vinifera*.

Ubiquitin-protein hydrolases are involved in the processing of ubiquitinated proteins. Ubiquitination in plant cells modulates signaling mediated by PAMP receptors and leads to the accumulation of NB-LRR receptors (Furlan et al., 2012). In Mgaloblishvili, the ubiquitination process appeared to be activated, upregulating genes encoding for RING H2-type E3 ligases (Toffolatti et al., 2018a), activated in response to biotic and abiotic stresses and involved in ubiquitination (Mazzucotelli et al., 2006).

Protein phosphorylation, by a combined action of protein kinases and phosphatases, is a rapid post-translational control mechanism in the response to environmental stimuli, such pathogen elicitors, playing a major role in signal transduction pathways (Friso and van Wijk, 2015). Some DNA-binding proteins, with phosphatase activity, are able to bind defense-related genes and take part in their transcriptional regulation (i.e., DBP1 controlling transcription of the defense-related CEVI1 gene in *A. thaliana* during plant–virus interaction) (Carrasco et al., 2003). MDP-1 and MDP-1-like genes can be involved in the transcriptional regulation of some defense-related genes in the *V. vinifera*-*P. viticola* interactions.

The locus named li_T_C_chr16_21398409 (*Rpv31*) was annotated in linkage group including several rust resistance kinase Lr10-like genes. As already described above, Mgaloblishvili could recognize *P. viticola* through specific NB-LRR receptors, such as several Lr10 genes. It was demonstrated the Lr10 confers enhanced resistance to *Puccinia triticina* in *Triticum aestivum* (Feuillet et al., 2003). Frequently, NB-LRR genes occur in clusters. In *Vitis*, the *Rpv12* locus accounts for 13 NB-LRR genes in a region of about 600 kb and it is part of a cluster of 46 NB-LRRs in the upper arm of chromosome 14 (Venuti et al., 2013). In our study, three Lr10-like genes (LOC100251517, LOC100256646, LOC100242248), spanning a region of about 47 kb on chromosome 16, appeared to be associated with the *P. viticola* resistance trait. Also, these three Lr10-like genes are part of a wider region, including a higher number of NB-LRR genes. In Toffolatti et al. (2018a), seven Lr10-like genes were differentially expressed (with a log2 fold-change value higher than 2) in Mgaloblishvili after *P. viticola* inoculation. Among them, four are located on chromosome 16, spanning a region of about 6 Mb. GWA results corroborate the involvement of these receptors in triggering the plant response. Indeed, during the infection process *P. viticola* has shown the expression of numerous different cytoplasmic and apoplastic effectors (Toffolatti et al., 2020) and their interaction with the NB-LRR receptors of the plant should be further investigated. Since no hypersensitive response (HR) was observed in the Georgian resistant accessions, due to absence of co-evolution with the pathogen, the involvement of the effector receptor Lr10 could be associated with an effector-triggered immunity not associated with HR. Indeed, HR is not always occurring in ETI (Jones and Dangl, 2006).

## Conclusion

In this study, for the first time in *V. vinifera*, GWAS was used to identify loci associated with the resistance to *P. viticola* attack. The analysis provided evidence of three novel resistant loci (*Rpv29*, *Rpv30*, and *Rpv31*) in a panel of Georgian accessions, that they could be utilized for further genetic and breeding studies to select genotypes showing resistance to *P. viticola* infection. The three loci were found to co-locate in genomic regions enriched of genes associated with plant defense mechanism against biotic stress, suggesting both PAMP-triggered immunity and ETI-HR free response. Nevertheless, this hypothesis should be validated, by functionally characterize the candidate genes. Functional genomics approaches, such as CRISPR-based (Clustered Regularly Interspaced Short Palindromic Repeats) or RNA interference technologies, can help to functionally validate the candidate genes and, thus, to investigate which gene(s) is essential for resistance to *P. viticola* infection.

The great advantage provided by sources of resistance in *V. vinifera* germplasm, compared to the *non-vinifera* one, lies in the possibility to obtain crosses with cultivated varieties showing a good resistance level against a specific pathogen and, at the same time, able to provide a product free from the unpleasant characteristics usually imparted by the American vines, first of all the foxy flavor of the grapes. The discovery of resistant sources in the *V. vinifera* background is crucial to exploit favorable alleles already present in a germplasm, coupling at the same time good resistance to pathogen and good agronomic traits. Indeed, Caucasian accessions show very attractive characteristics for high-quality production also in the perspective of the climate change, such as late ripening, medium-size berries, avoidance of excessive sugar accumulation, smooth tannin and ability to maintain good level of acidity.

## Data Availability Statement

The datasets presented in this study can be found in online repositories. The names of the repository/repositories and accession number(s) can be found in the article/Supplementary Material.

## Author Contributions

GD, ST, OF, DM, and PB conceived the study. GM and NB performed phenotypical analysis. MS performed SNP profiling and analyzed the data. MS, GD, and ST wrote the manuscript. All authors approved the final version of the article.

## Conflict of Interest

The authors declare that the research was conducted in the absence of any commercial or financial relationships that could be construed as a potential conflict of interest.

## Footnotes

1 http://www.oiv.int/

## Supplementary Material

Supplementary Figure S1 Box-plot distribution of the I%I recorded by each grapevine accession, belonging to a Mgaloblishvili self-pollinated population (84) and Georgian germplasm population (48), following *P. viticola* inoculation. Resistant accessions are highlighted in red.

Supplementary Table S1 List of grapevine accessions phenotyped for their resistance/susceptibility to *P. viticola* infection and genotyped by the Vitis18kSNP genotyping array. Phenotype column reports the resistance (1) or susceptibility to *P. Viticola* infection and resistance levels. The accessions showing a percentage of infection lower than 25%, in the three years of sampling (2017, 2018, and 2019), were considered resistant. Phenotypical evaluations were performed in triplicate.

Supplementary Table S2 SNP profiles of 132 grapevine accessions, belonging to a Mgaloblishvili self-pollinated population (84) and Georgian germplasm population (48), genotyped at 18 k loci. Row data were filtered based on SNP call quality (p50GC) < 0.54, GenTrain score > 0.6, marker missing rate > 20% and minor allele frequency (MAF) > 5%, resulting in 12,825 SNP loci used for GWAS. “AA”: homozygous for dominant allele; “AB”: heterozygous for dominant allele; “BB”: homozygous for recessive allele; “NC”: missing data.

Supplementary Table S3 Ancestry values at *K* = 3 detected for SNP profiles of 132 grapevine accessions, belonging to a Mgaloblishvili self-pollinated population (84) and Georgian germplasm population (48), genotyped at 18 k loci.

Supplementary Table S4 List of candidate genes in a window of 100 kb upstream and downstream the three SNPs associated to resistance trait to *P. viticola* infection.

Supplementary Table S5 Allele information on SNP loci detected in a region spanning 100 kb upstream and downstream the three loci associated (highlighted in gray) to the *P. viticola* resistance. In red: resistant genotypes.
