# Supplementary figures and images for: Rpv29, Rpv30 and Rpv31: Three Novel Genomic Loci Associated With Resistance to Plasmopara viticola in Vitis vinifera
Source: Front Plant Sci. 2020 Oct 8;11:562432. doi: 10.3389/fpls.2020.562432 (PMC7583455; doi:10.3389/fpls.2020.562432)

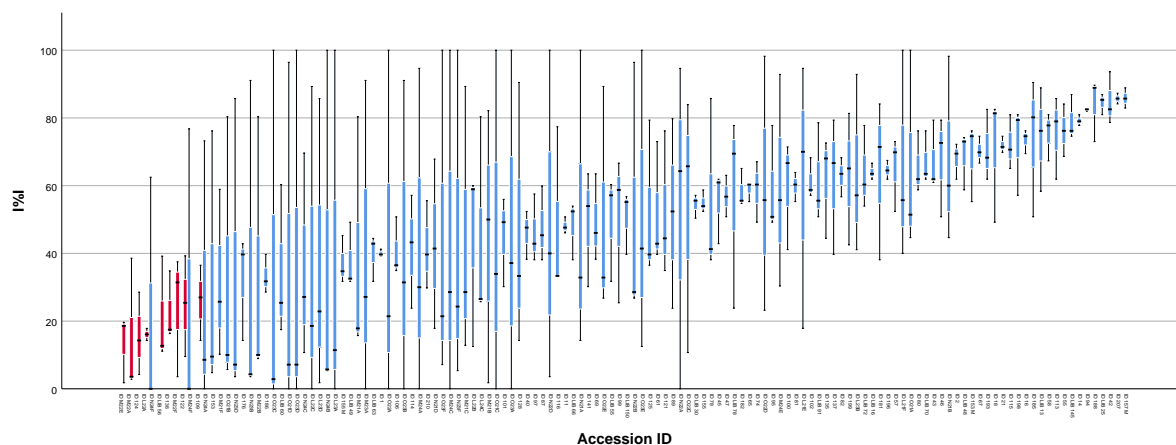

Supplement: Supplementary Figure S1 — Box-plot distribution of the I%I recorded by each grapevine accession, belonging to a Mgaloblishvili self-pollinated population (84) and Georgian germplasm population (48), following P. viticola inoculation. Resistant accessions are highlighted in red. [file Image_1.pdf]
