# Supplementary material for: Rpv29, Rpv30 and Rpv31: Three Novel Genomic Loci Associated With Resistance to Plasmopara viticola in Vitis vinifera
Source: Front Plant Sci. 2020 Oct 8;11:562432. doi: 10.3389/fpls.2020.562432 (PMC7583455; doi:10.3389/fpls.2020.562432)
Supplement: Supplementary Table S4 — List of candidate genes in a window of 100 kb upstream and downstream the three SNPs associated to resistance trait to P. viticola infection. [file Table_4.docx]

**Supplementary Table 4** List of candidate genes in a window of 100kb upstream and downstream the three SNPs associated to resistance trait to *P. viticola* infection.

| **Chromosome** | **Position from..to (bp)** | **Gene annotation** | **Protein description** |
| --- | --- | --- | --- |
| 14 | 21533965..21534998 | VIT_214s0006g03076 | uncharacterized protein |
|  | 21553355..21554075 | VIT_214s0006g03080 | uncharacterized protein |
|  | 21558910..21560892 | VIT_214s0006g03090 | probable cellulose synthase A catalytic subunit 8 [UDP-forming] |
|  | 21607164..21608222 | VIT_214s0006g03100 | uncharacterized protein |
|  | 21611382..21612188 | VIT_214s0006g03110 | acyl-CoA-binding domain-containing protein 3-like |
|  | 21612866..21742251 | VIT_214s0006g03120 | HEAT repeat-containing 5B protein |
|  | 21742614..21743660 | VIT_214s0006g03180 | probable carboxylesterase 17 |
|  | 21746749..21749225 | VIT_214s0006g03190 | plant cadmium resistance 4 protein |
| 3 | 16125132..16127240 | VIT_203s0017g00380 | magnesium-dependent phosphatase 1-like |
|  | 16137049..16137935 | VIT_203s0017g00390 | MADS-box protein JOINTLESS-like |
|  | 16166870..16167220 | VIT_203s0017g00396 | ubiquitin carboxyl-terminal hydrolase 21 |
|  | 16190988..16192049 | VIT_203s0017g00410 | magnesium-dependent phosphatase 1 |
|  | 16192103..16206554 | VIT_203s0017g00420 | uncharacterized protein |
|  | 16235590..16246518 | VIT_203s0017g00440 | uncharacterized protein |
|  | 16246761..16247367 | VIT_203s0017g00450 | MADS-box protein JOINTLESS-like |
|  | 16314218..16334275 | VIT_203s0017g00460 | inositol transporter 1 |
| 16 | 21309145..21309516 | VIT_216s0050g02800 | uncharacterized protein |
|  | 21312366..21319643 | VIT_216s0050g02810 | uncharacterized protein |
|  | 21379089..21381377 | VIT_216s0148g00010 | rust resistance kinase Lr10-like |
|  | 21381945..21385100 | VIT_216s0148g00020 | leaf rust 10 disease-resistance locus receptor-like protein kinase (like 2.5) |
|  | 21421886..21425581 | VIT_216s0148g00030 | rust resistance kinase Lr10-like |
|  | 21498263..21501414 | VIT_216s0148g00040 | rust resistance kinase Lr10-like |
